# Pathogenesis of Viral Hepatitis-Induced Chronic Liver Disease: Role of Extracellular Vesicles

**DOI:** 10.3389/fcimb.2020.587628

**Published:** 2020-11-10

**Authors:** Hong Kiat Lim, Gary P. Jeffrey, Grant A. Ramm, Carolina Soekmadji

**Affiliations:** ^1^ Hepatic Fibrosis Group, Department of Cellular and Molecular Biology, QIMR Berghofer Medical Research Institute, Brisbane, QLD, Australia; ^2^ Faculty of Health and Medical Sciences, University of Western Australia, Perth, WA, Australia; ^3^ Sir Charles Gairdner Hospital, Nedlands, Hepatology Department and Liver Transplant Service, Perth, WA, Australia; ^4^ Faculty of Medicine, The University of Queensland, Brisbane, QLD, Australia

**Keywords:** extracellular vesicles, chronic liver disease, hepatic fibrosis, therapy, biomarker

## Abstract

Extracellular vesicles are encapsulated lipid nanoparticles secreted by a variety of cell types in living organisms. They are known to carry proteins, metabolites, nucleic acids, and lipids as their cargoes and are important mediators of intercellular communication. The role of extracellular vesicles in chronic liver disease has been reported. Chronic liver disease such as viral hepatitis accounts for a significant mortality and morbidity burden worldwide. Hepatic fibrosis has been commonly associated with the chronic form of viral hepatitis, which results in end-stage liver disease, including cirrhosis, liver failure, and carcinoma in some patients. In this review, we discuss the potential role of extracellular vesicles in mediating communication between infectious agents (hepatitis B and C viruses) and host cells, and how these complex cell-cell interactions may facilitate the development of chronic liver disease. We will further discuss how understanding their biological mechanism of action might be beneficial for developing therapeutic strategies to treat chronic liver disease.

## Introduction

Extracellular vesicles (EV), first described over four decades ago (Chargaff and West, 1946; Wolf, 1967), have now gained recognition as an important mediator of intercellular communication in chronic liver disease (CLD) (Ramakrishnaiah et al., 2013; Hirsova et al., 2016; Devhare et al., 2017; Banales et al., 2019). These membrane-bound nanoparticles are secreted by a variety of cell types in a living organism and are known to carry cargoes such as proteins, metabolites, nucleic acids, and lipids, which mediate complex cell-cell communications. The specific nature of EV-derived cargo has led to an immense interest in using EV as a tool for disease diagnosis and as a target for therapeutic intervention (Banales et al., 2019; Soekmadji et al., 2020).

The CLD is an umbrella term used for reference to any pathological condition where progressive destruction of liver tissue occurs over 6 months or more. CLD is a significant health concern worldwide; chronic hepatitis C, chronic hepatitis B, alcoholic or non-alcoholic fatty liver disease, and autoimmune hepatitis account for a significant global mortality and morbidity burden. It is estimated that about 844 million people are living with CLD (2017), and at least 2 million deaths per year are associated with CLD globally (Byass, 2014). Cell-cell interactions amongst cell types such as liver progenitor cells (LPC), hepatic stellate cells (HSC), and Kupffer cells have been implicated in the development of liver fibrosis and CLD, which eventually progress into an end-stage liver disease including cirrhosis, liver failure and carcinoma (Dwyer et al., 2014; Pozniak et al., 2017). Recently, EVs have been shown to mediate these complex cell interactions among these cell types in the liver, particularly in the context of CLD, which shed light on their potential roles in the development of this disease (Deng et al., 2017).

This review discusses the potential role of EVs in mediating communication between infectious agents and host cells, and how EV-mediated cell-cell interactions facilitate the development of liver disease. We will further discuss how understanding their biological mechanism of action might be beneficial for developing therapeutic strategies to treat chronic liver disease.

## Liver Structure and Function

**Figure 1:**
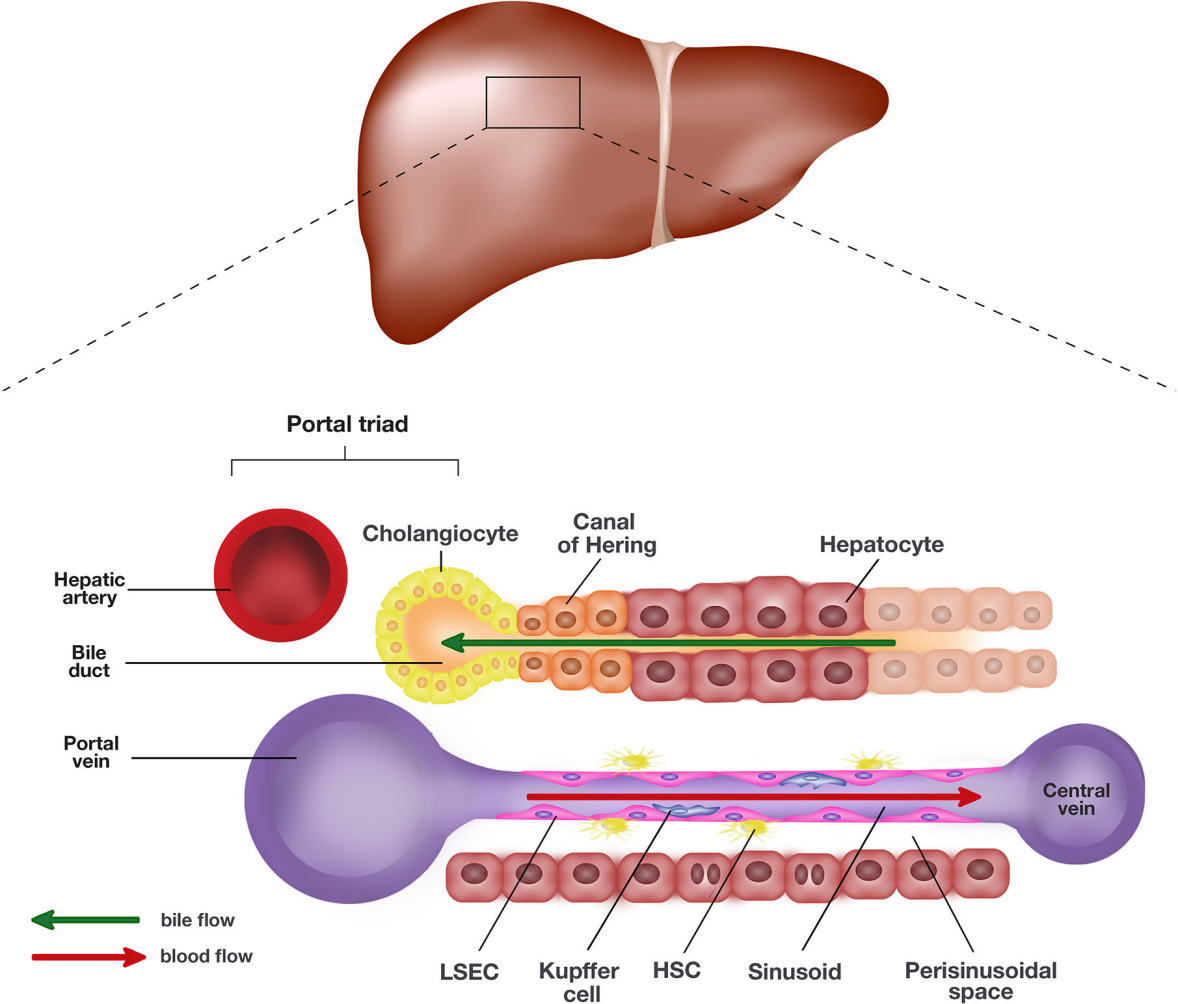
Schematic representation of the liver and the single end of its lobule. The portal triad comprising the hepatic artery, bile duct, and portal vein sits at each end of the hepatic lobule. Blood collected from the portal vein and hepatic artery flows toward the central vein through the hepatic sinusoids lined by liver sinusoidal endothelial cells (LSEC) and hepatocytes. The hepatocytes and LSECs are separated by the perisinusoidal space, also known as space of Dissé where hepatic stellate cells (HSC) are located. The hepatocytes produce bile which empties into the bile duct lined by cholangiocytes. The canal of Hering is positioned in the junctional region between cholangiocytes and hepatocytes, where liver progenitor cells (LPC) are proposed to originate.

The liver is the largest visceral organ in the body, making up an estimated 2–5% of the adult body weight with roughly 10% of the body’s blood flowing through at any one time (Vekemans and Braet, 2005). The liver performs a myriad of homeostatic roles associated with metabolism, digestion, immunity, and the endocrine system. Microscopically, the liver is composed of two main cell types, parenchymal and non-parenchymal cells (Trefts et al., 2017). Parenchymal cells, including hepatocytes and cholangiocytes, form the majority of cell types in the liver. Hepatocytes, along with the liver sinusoidal endothelial cells (LSEC), line the sinusoids and are the primary epithelial cells of the liver (Trefts et al., 2017). Hepatocytes and LSECs are separated by the perisinusoidal space, also known as space of Dissé. Cholangiocytes line the bile ducts and are involved in the production and modification of bile composition (**Figure 1**).

In the injured liver, a unique subset of stem-like cells are induced termed hepatic or liver progenitor cells (LPC), also described as oval cells in rodents, which have the potential to reconstitute liver mass by differentiation into hepatocytes or cholangiocytes (Tirnitz-Parker et al., 2010; Köhn-Gaone et al., 2016). LPCs are proposed to originate in the canal of Hering in relatively small numbers at steady state but quickly expand through rapid proliferation following chronic hepatic injury (Dwyer et al., 2014). While their origin remains controversial, recent lineage tracing studies have reported that activated Sox9^+^-ductal cells proliferate and differentiate into liver progenitor cells (LPCs) after sustaining chronic hepatic insults (Furuyama et al., 2011).

Nonparenchymal cells of the liver comprise liver myofibroblast precursors called hepatic stellate cells (HSC), resident liver macrophages or Kupffer cells and LSECs. HSCs which usually reside in the perisinusoidal space are liver-specific mesenchymal cells rich in vitamin A (Higashi et al., 2017). They exist at the ratio of 3.6 to 6 cells per 100 hepatocytes in human liver, and their primary function in the normal liver appears to involve vitamin A storage (Moreira, 2007). Studies have also reported their regulatory role in regulating hepatic blood flow and portal venous pressure at steady state (Geerts, 2001). LSECs are specialized endothelial cells, which form the highly fenestrated sinusoidal endothelium needed for the exchange of fluid, nutrients, and solutes between the sinusoidal blood and hepatocytes (Ni et al., 2017). They are highly endocytic and have a well-developed clathrin-mediated endocytosis system (Simon-Santamaria et al., 2010). Finally, Kupffer cells are specialized macrophages in the liver. They form part of the reticuloendothelial system and are involved in clearing senescent cells and pathogens such as bacteria and viruses. They are one of the key players in hepatic immunity.

## Pathophysiology of Chronic Liver Disease

While the liver represents an organ with enormous regenerative potential, chronic hepatic insults from pathogens, metabolic insults, and other toxic agents can lead to the development of CLD where the ability of the liver to heal and regenerate diminishes as a consequence of hepatic scarring (fibrosis) and eventually results in deterioration of liver function. The development of CLD is a complex multifactorial process involving many different cell types. Following a hepatic insult, the liver attempts to repair the injured tissue through the normal wound healing process. Paracrine stimulatory signals, including inflammatory mediators, from other cell types such as LPCs, LSECs, Kupffer cells, and hepatocytes within the liver microenvironment activate quiescent dormant HSCs to proliferate and migrate into the primary site of insults. These activated α-smooth muscle actin- (SMA) and collagen type I-expressing HSCs transdifferentiate into myofibroblasts, which produce collagen and extracellular matrix needed for the wound healing process (Higashi et al., 2017). They rapidly lose their ability to store Vitamin A, causing the amount of Vitamin A within the cell to decrease as it divides and distributes Vitamin A-lipid droplets into two daughter cells (Higashi et al., 2005). In CLD, these HSC-derived myofibroblasts contribute to excessive deposition of collagen and extracellular matrix in response to chronic liver injury; thus they are responsible for hepatic fibrosis in a variety of different CLDs in adults including chronic hepatitis C virus (HCV), alcoholic liver disease, non-alcoholic fatty liver disease (Friedman, 2008), liver cancer (Bridle et al., 2001) haemochromatosis (Ramm et al., 1997), and in pediatric liver disease such as biliary atresia (Ramm et al., 1998) and cystic fibrosis-associated liver disease (Lewindon et al., 2002).

LPCs can rapidly proliferate and differentiate in response to liver injury in a process called the ductular reaction. However, the role of LPCs in liver regeneration and repair seems to be restricted to chronic injury where the replication of mature hepatocytes has been impaired, or the hepatic microenvironment has been substantially changed (Dollé et al., 2010; Best et al., 2013). Clouston *et al*. showed that inflammatory cytokines such as interferon (IFN)-γ inhibited proliferation of hepatocytes, resulting in an expansion of LPCs (Clouston et al., 2005). One possible mechanism for LPC expansion involves tumor necrosis factor-like weak inducer of apoptosis (TWEAK) and its receptor, fibroblast growth factor-inducible 14 (Fn14)-signaling (TWEAK/Fn14 signaling) (Dwyer et al., 2014). Binding of TWEAK secreted by macrophages or natural killer (NK) cells to Fn14 expressed on the surface of LPCs activates the downstream NFκB signaling pathway, which switches the genes involved in proliferation on, leading to the expansion of LPCs (Tirnitz-Parker et al., 2010; Viebahn et al., 2010; Bird et al., 2013).

Interactions between HSCs and LPCs have been shown to drive hepatic fibrogenesis in CLD. Notch signaling has previously been implicated in the biliary specification of Notch1/Notch2^+^ LPCs through interactions with Jagged1^+^ myofibroblasts (Boulter et al., 2012). The study demonstrated a decrease in expression of biliary genes in LPCs when Notch inhibitor was used in co-cultures of LPCs and HSCs. Interestingly, another study demonstrated impairment in the differentiation of LPCs into cholangiocytes when Notch2 liver-specific knockout mice were treated with 3,5-diethoxycarbonyl-1,4-dihydrocollidine (DDC) (Fiorotto et al., 2013). Previous studies in this field identified a novel regulatory mechanism of HSC/LPC crosstalk involving lymphotoxin beta (LTβ) and its receptor, LTβR (Ruddell et al., 2009; Tirnitz-Parker et al., 2014). LTβ expression has been shown to increase in several animal models of chronic liver injury (Lowes et al., 2003; Akhurst et al., 2005; Knight et al., 2005; Lee et al., 2005; Dwyer et al., 2014). One study found that LTβR^−/−^ animals fed with choline-deficient, ethionine-supplemented (CDE) diet to induce biliary fibrosis had decreased levels of inflammatory cytokines, which paralleled the reduction in fibrosis and activated HSC numbers, compared to the wild-type animals (Ruddell et al., 2009). Moreover, LPCs were shown to express LTβ, while LTβR expression was detected on quiescent/activated HSCs in that study, further suggesting the possible role of LTβ/LTβR signaling in HSC/LPC crosstalk (Ruddell et al., 2009).

In CLD, interactions between HSC and LSEC have also been previously reported (Horn et al., 1987; DeLeve et al., 2008b). LSECs undergo phenotypic changes in a process called capillarization prior to HSC activation and fibrosis (Horn et al., 1987; DeLeve et al., 2008b). Activated LSECs defenestrate and form a continuous basement membrane liken to the phenotype of capillaries in order to restrict the movement of toxic molecules which would otherwise be harmful to the liver (Deleve et al., 2008a; Ni et al., 2017). Unfortunately, this safety mechanism also prevents an adequate exchange of solute or fluid between sinusoids and hepatocytes, which further exacerbates the fibrotic process in the liver (Bartneck et al., 2014). While healthy LSECs prevent activation of HSC and initiate changes of HSC from an activated to a quiescence state, capillarized LSECs promote HSC activation (Deleve et al., 2008a).

## Viral Hepatitis as a Cause of Chronic Liver Disease

Viral hepatitis is one of the most common causes of CLD affecting close to 397 million individuals worldwide with both hepatitis B virus (HBV) and HCV infection cases combined (Trepo et al., 2014; Westbrook and Dusheiko, 2014; Ferri et al., 2016). There are five main types of hepatitis viruses, including types A, B, C, D, and E, of which, types B and C remain prevalent globally, albeit differing in the geographical distribution of disease prevalence. Infections associated with hepatitis types B, C, D, and E viruses cause CLD at varying rates with type E being the rarest, although co-infection of type B is still required for type D to become chronic. While hepatitis viruses primarily infect hepatocytes, some studies have reported binding of hepatitis types C viruses to other cell types such as peripheral blood mononuclear cells (PBMC) (El-Awady et al., 2005; Yamada et al., 2005), which raises the possibility that other cell types could play a more significant role in the pathogenesis of viral hepatitis. Most of the pathophysiology of viral-induced CLD is attributed to the exacerbated host immune response to the virus rather than the viral replication in the cells, although more work has to be done to clarify the mechanisms involved (Nakamoto et al., 1998; Buchmann et al., 2013; Ringelhan et al., 2017). This review will focus on HBV and HCV, given their high degree of clinical relevance in CLD and disease prevalence.

HBV, an enveloped partially double-stranded DNA virus, belongs to the Hepadnaviridae family, genus Orthohepadnavirus (Sekiba et al., 2018). The infectious viral particles are double-shelled and spherical. They consist of an outer lipid envelope embedded with hepatitis B surface antigens (HBsAg), and a nucleocapsid which comprises hepatitis B core antigens (HBcAg), viral polymerase, and DNA genome (Liang, 2009). The HBV genome is a 3.5kb long relaxed circular DNA (rcDNA), consisting of four overlapping reading frames, which encodes envelope proteins, structural core, viral polymerase/reverse transcriptase, and regulatory x protein (HBx) (Liang, 2009). HBV first enters the cells using the sodium taurocholate co-transporting polypeptide receptor (Yan et al., 2012; Ni et al., 2014; Tong and Li, 2014). After entry, the virion un-coats in the cytoplasm, allowing rcDNA to be transported into the host nucleus where rcDNA is converted into covalently closed circular DNA (cccDNA) (Grimm et al., 2011). The host RNA polymerase II transcribes cccDNA into viral messenger RNA (mRNA) transcripts containing viral pregenomic RNA (pgRNA), which are then re-exported back into the cytoplasm of the host cell for translation of viral proteins (Rajbhandari and Chung, 2016). Assembly of nucleocapsids occurs in the cytoplasm where pgRNA is co-packaged with viral polymerase/reverse transcriptase for reverse transcription of pgRNA into rcDNA. Mature nucleocapsids are enveloped through the endoplasmic reticulum (ER) and Golgi apparatus before secretion from the host cell *via* exocytosis (Rajbhandari and Chung, 2016). While the majority of pgRNA is reverse transcribed into rcDNA, a small proportion (10%) gets synthesized into double-stranded linear DNA (dslDNA) which can integrate into the host genome. However, unlike retroviruses, integration of HBV DNA is not essential for viral replication (Sekiba et al., 2018).

HBV is known to cause both acute and chronic forms of hepatitis. However, it is estimated that 10% of acute infection progresses to chronic disease (McKeating et al., 2018). The clinical manifestations of acute hepatitis include flu-like symptoms, dark urine, and jaundice, although some cases may appear asymptomatic. While the majority of HBV-infected people recover from the infection completely, some people remain chronically infected with HBV. In chronic hepatitis, a persistent unproductive immune response to HBV is responsible for substantial necroinflammation of the liver. It has been shown that T helper (Th) 2 type cytokines such as IL-4 and IL-10 are associated with persistent HBV infections resulting in more severe liver damage (Lee et al., 1999). Indeed, the importance of a protective T helper (Th) 1 rather than a tolerant Th2 immune response in clearing HBV has been demonstrated by several studies (Marinos et al., 1995; Maini et al., 2000). Akbar et al**. demonstrated the importance of IFN-γ in controlling HBV infections by showing a reduction in HBV DNA in the liver and sera after dendritic cells were treated with Th1 type cytokine; IFN-γ (Akbar et al., 1996). In addition to host immune response, studies have reported the involvement of viral component HBx in the progression of chronic HBV. It was first shown by Lee et al**. that HBx antigen was able to inhibit CD8^+^ T cell response by reducing the production of IFN-γ and inducing apoptotic program in CD8^+^ T cells (Lee et al., 2010). Other findings have also shown that HBx induces innate pro-inflammatory IL-6, IL-8, and TNF-α but often not at the level sufficient for viral clearance in chronic HBV (Mahé et al., 1991; Lara-Pezzi et al., 1998; Lee et al., 1998). In particular, it has been shown that IL-6 initiates a switch from acute to chronic inflammation by recruiting monocytes to the inflammation sites (Gabay, 2006). Furthermore, HBx was found to activate and promote the proliferation of HSCs (Martín-Vílchez et al., 2008; Bai et al., 2012).

While it is widely accepted that a substantial number of chronic HBV-associated liver cirrhosis cases develop hepatocellular carcinoma (HCC) as an end-stage complication of the infection (El-Serag and Mason, 1999; Block et al., 2003; El-Serag and Rudolph, 2007), little is known about the process in which the malignant transformation occurs. Persistent hepatic necroinflammation as a result of viral-host immune interaction is, however, recognized as the primary driver of HCC development (Ringelhan et al., 2017; Chen and Tian, 2019). Interestingly, as opposed to HCV-driven HCC, which develops mainly in the presence of cirrhosis, only about 20% of HCC driven by chronic HBV infection occur with cirrhosis (Chayanupatkul et al., 2017). Indeed, the development of HCC in HBV-infected individuals in the absence of inflammation shed light on alternative mechanisms for tumorigenesis. Epidemiology data revealed that 85–90% of HBV-associated HCCs contained HBV DNA integrated into the host genome (Minami et al., 2005), raising the possibility that the integrated viral DNA might be involved in HCC development. Integration of HBV viral DNA was previously shown to activate expression of oncogenes such as cyclin E1 (CCNE1), telomerase reverse transcriptase (TERT), and mixed-lineage leukemia 4 (MLL4) (Sung et al., 2012). Moreover, genetic instability leading to chromosomal translocation and accumulation of genetic mutations after integration of viral HBx into the host genome has been reported (Lee and Rho, 2000; Bonilla Guerrero and Roberts, 2005; Feitelson and Lee, 2007).

HCV is an enveloped positive-sense single-stranded RNA virus belonging to the Flaviviridae family, genus hepacivirus. The HCV virions contain E1 and E2 glycoproteins within the viral envelope that surrounded the core protein and nucleocapsid. The HCV genome is 9.6kb long, consisting of two untranslated regions (UTR) 5’-UTR and 3’-UTR and an open reading frame (ORF). The ORF encodes a polyprotein, which is further processed into various viral proteins including core protein, E1, E2, p7, NS2, NS3, NS4A, NS4B, NS5A, and NS5B. The life cycle of HCV, while only partially elucidated, starts with the attachment of HCV to a host receptor. Several host receptors, including lipoprotein (LDL) receptor, scavenger receptor B1 (SR-B1), and CD81 tetraspanin (which is also an EV marker) (Théry et al., 2018) have been proposed to be involved. Once bound to its receptor, HCV-receptor complex is internalized through clathrin-mediated endocytosis, releasing the nucleocapsid into the cytoplasm. Viral genomic RNA is freed from the nucleocapsid after uncoating, which then undergoes genomic replication *via* a negative-sense RNA intermediate and translation of polyproteins at the ER for further post-translational processing. Assembly and maturation of virion take place in the ER and Golgi apparatus before releasing from the host cell by exocytosis.

About 80% of acute HCV cases develop into chronic HCV infection due to the inability of the host immune system in clearing the virus (McKeating et al., 2018). Several mechanisms responsible for the persistence of HCV in the host have been reported. First, the error-prone and high replicating characteristic of HCV RNA polymerase results in a high acquisition of mutations in the viral genome, leading to the generation of many HCV quasispecies with mutated epitopes, which cannot be recognized by cytotoxic CD8^+^ T cells (Irshad et al., 2013). Consequently, the cell-mediated cytotoxic killing of infected host cells is prevented, allowing the viruses to persist (Irshad et al., 2013). Moreover, persistent antigenic stimulation in chronic infection results in T cell exhaustion, which involves a loss of virus-specific effector T cells and increased expression of inhibitory molecules such as PD-1 (Penna et al., 2007; Radziewicz et al., 2007). For example, Wedemeyer *et al*. reported defective IFN-γ production and proliferative capacity with HCV-tetramer^+^ T cells in patients with chronic HCV infection (Wedemeyer et al., 2002). Second, there is evidence that HCV proteins drive Fas-mediated apoptosis of virus-specific immune cells while promoting infiltration of peripheral T cells into the liver (Soguero et al., 2002). It is thought that the apoptosis of T cells in the liver might be partially responsible for liver injury mediated by inflammation (Soguero et al., 2002). Third, it has been reported that HCV E2 glycoprotein suppresses the non-specific cytotoxicity function in NK cells, allowing HCV to persist in affected patients (Crotta et al., 2002). As with HBV infections, persistent hepatic necroinflammation in chronic HCV infections creates a microenvironment which favors the development of cirrhosis and malignancies. It has been reported that CD8^+^ T cells and NK cells are directly involved in the pathogenesis of HCV related liver cirrhosis and HCC (Cooper et al., 1999; Karidis et al., 2015; Khatun and Ray, 2019).

## The Role of Extracellular Vesicle(s) in the Pathogenesis of Viral Disease

The extracellular vesicle (EV) is a collective term for “particles released from cells that are delimited by a lipid bilayer and cannot replicate” (Théry et al., 2018). While EVs have been previously categorized into exosomes, microparticles, and apoptotic bodies, what constitutes each subtype has been ambiguous. Since no consensus has been reached on their specific markers, this review will refer to EV subtypes based on the recommendations from the International Society for Extracellular Vesicles (ISEV) (Théry et al., 2018). In terms of size, EVs can be defined as small (<100 or 200nm) and medium/large (>200nm) EVs (Théry et al., 2018). Small EVs are highly enriched in a class of membrane proteins called tetraspanins such as CD9, CD63, CD81, as well as cytosolic proteins TSG101 and Alix. These proteins also play diverse roles in cell biology and physiology (Andreu and Yáñez-Mó, 2014; Lozano-Andrés et al., 2019). In addition, EVs can be categorized based on their biochemical composition and conditions or cell where they originate (Théry et al., 2018).

Biogenesis of EVs differs among exosomes, microparticles and apoptotic bodies and has been extensively reviewed elsewhere (Akers et al., 2013; Schorey et al., 2015; Abels and Breakefield, 2016; Hirsova et al., 2016). In general, exosomes are described as vesicles that are formed by inward budding at the multivesicular endosomes. Multivesicular endosomes destined for degradation or exocytosis will either fuse with lysosomes for degradation of their contents, or with the plasma membrane for exocytosis (Abels and Breakefield, 2016). Exosome biogenesis involves either endosomal-sorting complex required for transport (ESCRT)-dependent or independent mechanisms. ESCRT is a type of multi-subunit molecular machinery and comprises five complexes (ESCRT-0, I, II, III and VPS4) with specific roles assigned to each ESCRT complex (Hirsova et al., 2016). These complexes are involved in cargo recognition, recruitment, vesicle maturation, and secretion (Hirsova et al., 2016). ESCRT independent mechanism, on the contrary, is largely lipid raft and ceramide-based and was first described in oligodendroglial cells (Trajkovic et al., 2008; Colombo et al., 2014).

### The Potential Role of Extracellular Vesicles in Viral Infectious Disease

The association between viral particles and EVs is indicated by the presence of viral elements in EVs isolated from infected cells (**Table 1**). Early studies in human immunodeficiency virus (HIV) have demonstrated the presence of viral transactivating response (TAR) element RNA in EVs isolated by gel filtration Sephadex G-10 spin column and Nanotrap particle A for CD63^+^ vesicles from sera of HIV-1 infected patients and supernatants of cultured infected J1.1 cells respectively (**Table 1**) (Narayanan et al., 2013; Sampey et al., 2016). It was shown that these TAR RNA-containing EVs prevented apoptosis and enhanced viral replication in recipient cells, causing these cells to be more susceptible to HIV-1 infection (Narayanan et al., 2013). Their role was further confirmed by a study, which demonstrated decreased susceptibility of recipient cells to HIV-1 infection after the release of those EVs was inhibited (Sampey et al., 2016). Furthermore, HIV virulence factor, Nef detected in plasma EVs of HIV-infected patients using sucrose density gradient ultracentrifugation was shown to correlate with the low T cell counts in patients (**Table 1**) (Lee et al., 2016). Indeed, another study reported the induction of CD4^+^ T cell apoptosis *in vitro* by EV-associated HIV Nef (**Table 1**) (Lenassi et al., 2010). Like the EVs from HIV-infected cells, the EVs isolated from Epstein-Barr virus (EBV)-positive A-type lymphoblastoid cells contain biomolecules such as latent membrane protein 1 (LMP1) which was found to inhibit NK and T cell functions (**Table 1**) (Dukers et al., 2000; Flanagan et al., 2003). In the context of human papillomavirus (HPV), it was found that the amount of EVs secreted by HeLa cells increases when E6/E7 oncogene expression is inhibited (Honegger et al., 2013). Moreover, the inhibition of E6/E7 oncogene expression leads to a reduction in the levels of EV cargo survivin, a negative regulator of apoptosis, although the E6/E7 oncogene protein itself has not been detected within the EVs (Honegger et al., 2013). Further studies are needed in part due to the possibility of co-isolation of virus during EV preparation as shown by the presence of retrovirus and xenotropic murine leukemia virus-related virus in isolated EVs using ultracentrifugation (Knouf et al., 2009; Soekmadji et al., 2017). It should be noted that there is an overlap in the mechanism by which viruses and EVs may share in biogenesis, entry, and secretion mechanisms, as shown by enveloped viruses that co-utilize the host’s cellular machinery during infection (Nolte-’t Hoen et al., 2016). One cellular mechanism being investigated is the ESCRT which, as mentioned above, forms an integral part of the cellular endosomal system for EV biogenesis (Abels and Breakefield, 2016). Strickland *et al*. demonstrated inhibition of HIV-1 replication and budding through depletion of an ESCRT-I protein called TSG101 in the human embryonic kidney, HEK 293 cells (Strickland et al., 2017). TSG101 is also widely utilized as an EV marker (Soekmadji et al., 2013). Interestingly, knocking down TSG101 was shown to increase HBV production in human hepatoma, Huh-7 cells (Stieler and Prange, 2014). They demonstrated the importance of ESCRT-II subunits EAP30, EAP45, and EAP20 for HBV replication and showed that extracellular HBV was substantially reduced after knockdown of these ESCRT-II components (Stieler and Prange, 2014). Moreover, several studies have reported a role of tetraspanin EV markers in the life cycle of several viruses such as HIV-1 (Pelchen-Matthews et al., 2003; Fu et al., 2015), HCV (Bartosch et al., 2003; Fénéant et al., 2014), human papillomavirus (HPV) (Spoden et al., 2008; Scheffer et al., 2013), and influenza A virus (IAV) (Earnest et al., 2015; Earnest et al., 2017), which further suggests the potential convergence of EV and virus biogenesis pathways.

**Table 1:** Types of viral elements present in extracellular vesicles (EVs) released from cells infected by HIV or Epstein-Barr virus (EBV).

Causative agent	Cargo	Isolation process	EV characterization	Source	References
HIV^*^	TAR element RNA	Filtration and ultracentrifugation	Western blot (CD63, CD45, Hsp70^#^, and Alix) Transmission electron microscopy	Culture supernatant of Jurkat^^^ and J1.1^^^ cells	(Narayanan et al., 2013)
		ExoQuick™ reagent and gel filtration Sephadex G-10 spin column		Patient sera	
		Size-exclusion chromatography and Nanotrap particle A for CD63^+^ vesicles	Western blot (CD63 and Hsp70^#^)	Culture supernatant of Jurkat^^^ and J1.1^^^ cells	(Sampey et al., 2016)
	Nef protein	Ultracentrifugation	Western blot (CD9, CD63, and CD81)	Culture supernatant of plasmid Nef-transfected HeLa.CIITA^^^, Jurkat^^^ and SupT1^^^ cells	(Lenassi et al., 2010)
		Differential centrifugation and column-based bead isolation	Western blot (CD63 and CD81)	Culture supernatant of monocytes	(Lee et al., 2016)
		Sucrose density gradient ultracentrifugation		Patient sera	
EBV^*^	LMP1	Sequential centrifugation	Immunoelectron microscopy	Culture supernatant of DG-75^^^ and QIMR NB-B95-8^^^	(Flanagan et al., 2003)

*HIV, human immunodeficiency virus; EBV, Epstein-Barr virus.

^Jurkat; immortalized human T lymphocyte cell line, J1.1; HIV-1 lymphadenopathy associated virus (LAV) infected Jurkat E6 cell line, HeLaCIITA; immortalized human cervical cell line transfected with class II transactivator, SupT1; human T cell lymphoblastic lymphoma cell line, DG-75; Burkitt’s lymphoma cell line, QIMR NB-B95-8; EBV-positive A-type lymphoblastoid cell line.

^#^Hsp70 is not commonly used as EV markers despite being found in EVs (Théry et al., 2018).

### The Role of Extracellular Vesicles in Virus-Associated Chronic Liver Disease

**Figure 2:**
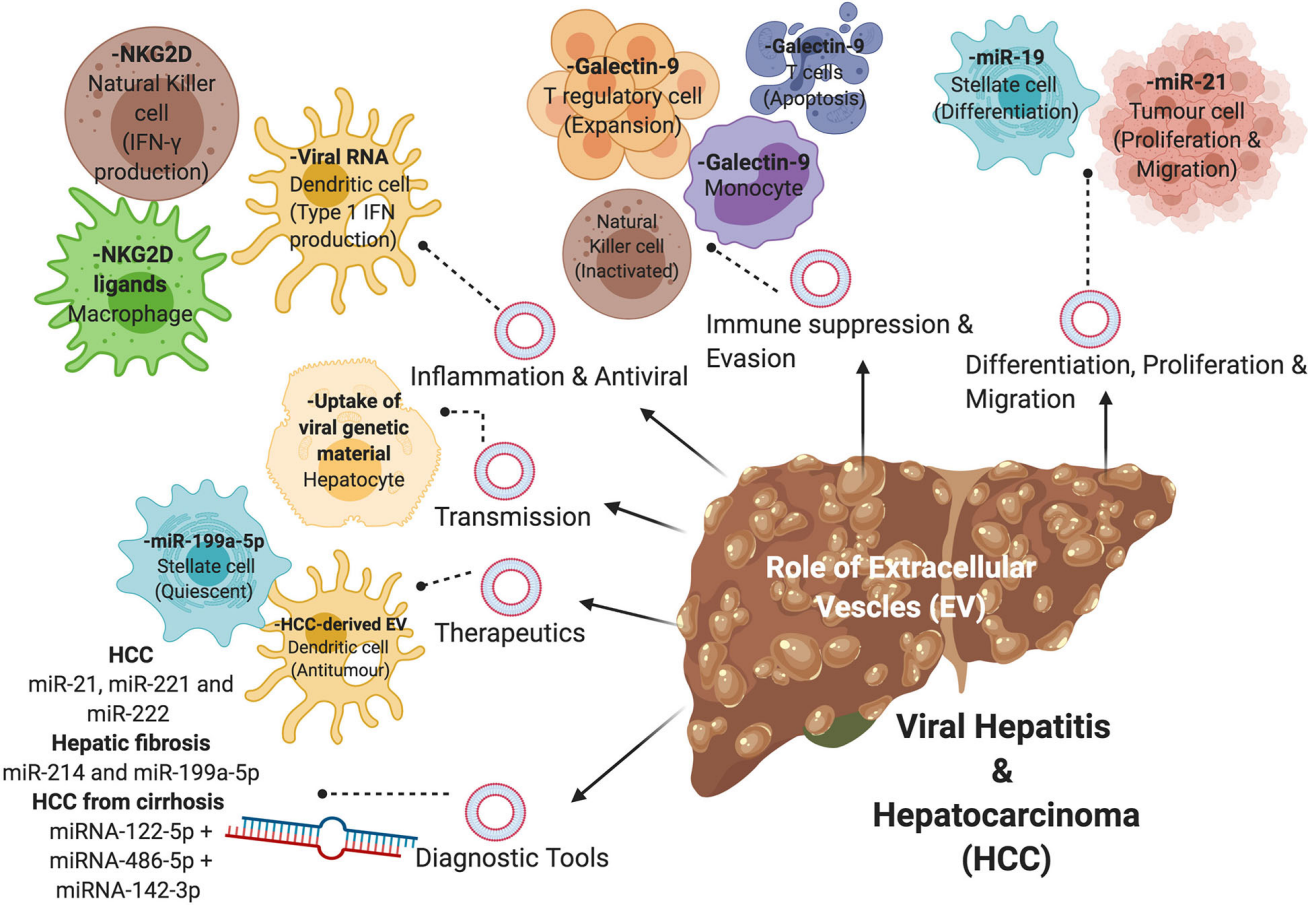
The role of extracellular vesicles (EVs) in viral-associated liver disease. EVs containing nucleic acids, proteins, or metabolites are involved in the disease progression of viral hepatitis and HCC *via* various mechanisms, including differentiation of hepatic stellate cells, proliferation and migration of tumor cells, immune suppression and evasion, inflammation and antiviral properties, and transmission of virions. EVs can also be used in the diagnosis and treatment of viral-associated liver disease.

While it remains unclear to what extent the similarities between viruses and EV biogenesis contributes to the disease progression of viral infections, it is possible that viruses may hijack the mechanism of EV biogenesis to increase infectivity and transmissibility due to their similarities with EVs. In addition, EVs could be utilized as a modulator of the host immune response against viruses. There is evidence to suggest a potential role of EVs in CLD associated with HBV and HCV infections (**Table 2**; **Figure 2**). The earliest strong evidence of EV involvement in viral-induced liver disease comes from studies that reported the presence of viral genetic materials co-isolated with EVs from culture supernatant using magnetic beads coated with antibodies specific for an EV marker CD81. Kouwaki *et al*. found that CD81^+^ EVs containing HBV-RNA isolated from plasmid pHBV-transfected HepG2 were able to induce expression of NKG2D ligands on macrophages, which has been known to simulate IFN-γ from NK cell (**Table 2**; **Figure 2**) (Kouwaki et al., 2016). Interestingly, a similar observation was made with HCV-RNA packaged EVs isolated from the culture supernatant of HCV-infected Huh-7.5.1c2 hepatocytes using sequential centrifugation, where these EVs were shown to activate plasmacytoid dendritic cells which produce type I IFN (**Table 2**; **Figure 2**) (Dreux et al., 2012). These observations suggest that EVs may be involved in the innate immune response against the virus. Further, while the contamination of EV preparation from pHBV-transfected HepG2 with free-floating viral genetic materials is possible, the contaminating viral genetic materials are unlikely to have affected the expression of NKG2D ligands on macrophages (**Table 2**; **Figure 2**). Although discrimination of virions and free-floating viral genetic materials from EVs has long been a challenging process, purer EV preparations could be obtained by immunoprecipitation using antibodies against EV specific markers (Zhang et al., 2019; Shahjin et al., 2020). As HBV DNA and proteins were found in EVs isolated using both differential centrifugation (Sukriti et al., 2019) and magnetic beads specific for CD63 (Yang et al., 2017) from the plasma/serum of patients with chronic HBV infection (**Table 2**), this strongly suggests that viral cargo could be present in EVs. These EVs containing HBV DNA were taken up by uninfected hepatocytes HepG2, leading to detectable HBV DNA levels (Sukriti et al., 2019) and expression of HBsAg and HBcAg in hepatocytes (Yang et al., 2017), which suggest a potential mechanism for viral transmission (**Table 2**; **Figure 2**). Indeed, findings by Sanada *et al*. demonstrating that HBV DNA-containing EVs isolated from HBV-infected primary hepatocytes of humanized mice were able to transmit to naïve hepatocytes, paralleled results reported in earlier studies (**Table 2**; **Figure 2**) (Sanada et al., 2016). This study also demonstrated the resistance of EVs to antibody neutralization, suggesting the possibility for EVs to act as a physical barrier between the infectious viral particles and the immune system, allowing viruses to establish persistence in chronic patients (Sanada et al., 2016).

**Table 2:** Involvement of extracellular vesicles (EVs) in chronic liver disease.

Causative agent or disease state	Origin of cargo	Cargo	Isolation process	EV characterization	Source	References
HBV^*^	Viral	HBV RNA	Total and CD81^+^ exosome isolation kits (Thermo Fisher Scientific)	Western blot (CD9, CD63, and CD81)	Culture supernatant of pHBV-transfected HepG2^^^ and Huh-7^^^ cells	(Kouwaki et al., 2016)
		HBsAg, HBeAg, and HBV DNA	Differential centrifugation	Flow cytometry (different size latex beads)	Patient platelet-free plasma	(Sukriti et al., 2019)
		HBV rcDNA and HBV RNA (HBx and HBs/p)	Ultracentrifugation and CD63-labeled Dynabeads^®^ positive selection (Life Technologies)	Flow cytometry (CD81) Electron microscopy	Patient sera	(Yang et al., 2017)
		HBV DNA	Sequential centrifugation and ultracentrifugation	Immunoprecipitation (CD9, CD63, and CD81) Stimulated emission depletion microscopy (CD81)	Culture supernatant of HBV-infected PXB^^^-cells	(Sanada et al., 2016)
		HBsAg, HBcAg, and HBV DNA	Ultracentrifugation and density gradient separation	Western blot (CD9 and CD63)	Culture supernatant of HepAD38^^^ cells	(Kakizaki et al., 2018)
	Host	miR-21 and miR-29a	Total and CD81^+^ exosome isolation kits	Western blot (CD63)	Culture supernatant of pHBV-transfected HepG2^^^ cells	(Kouwaki et al., 2016)
HCV^*^	Viral	HCV RNA	Sequential centrifugation	Western blot (CD63 and CD81)	Culture supernatant of HCV-infected Huh-7.5.1c2^^^ hepatocytes	(Dreux et al., 2012)
	Host	Galectin-9	ExoQuick™ method (System Biosciences)	–	Culture supernatant of HCV-infected Huh-7.5.l^^^ hepatocytes	(Harwood et al., 2016)
		miR-19a	Sequential centrifugation and ExoQuick™ method (System Biosciences)	–	Culture supernatant of HCV-infected IHH^^^ cells	(Devhare et al., 2017)
Hepatocellular Carcinoma	Host	miR-221 and miR-222	ExoQuick™ Exosome Precipitation Solution (System Biosciences)	Western blot (CD63, CD9, and calnexin^#^)	Patient sera	(Sohn et al., 2015)
		miR-21	Sequential centrifugation and ultracentrifugation	Western blot (CD63, CD81, and CD9) Transmission electron microscopy	Conditioned medium of HepG2^^^, Hep3B^^^, SNU-449^^^ and Huh-7^^^ cells	(Cao et al., 2019)
			Filtration and ExoQuick™ Exosome Precipitation Solution (System Biosciences)	Western blot (CD63 and Tsg101) Transmission electron microscopy	Patient sera	(Tanaka et al., 2013)
			Total Exosome Isolation Reagent (Invitrogen)	Western blot (CD63) Transmission electron microscopy	Patient sera	(Wang et al., 2014)
Hepatic Fibrosis	Host	miR-214	Sequential centrifugation	Transmission electron microscopy Zeta potential analysis and dynamic light scattering Western blot (CD9)	Conditioned medium of activated passage 6 mouse pHSC^^^	(Chen et al., 2014)
		miR-199a-5p	Sequential centrifugation	NanoSight nanoparticle tracking analysis Western blot (CD81)	Conditioned medium of activated passage 6 mouse pHSC^^^	(Chen et al., 2016)
		miR-122, miR-192, and miR-200b	Total Exosome Isolation Reagent (Thermo Fisher Scientific)	Western blot (CD63 and Tsg101)	Patient plasma	(Lambrecht et al., 2017)

*HBV, hepatitis B virus; HCV, hepatitis C virus.

^HepG2; human hepatocellular carcinoma cell line, Huh-7; human hepatocellular carcinoma cell line, Huh-7.5.1 or Huh-7.5.1c2; subclone of Huh-7 cell line with HCV strain JFH-1 subgenomic replicon, PXB; human primary hepatocytes from liver of humanized mice, HepAD38; HepG2 cell line with a stable integration of an HBV genome, IHH; immortalized human hepatocytes, Hep3B; human hepatocellular carcinoma cell line which contains an integrated HBV genome, SNU-449; human hepatocellular carcinoma cell line which contains an integrated HBV genome, pHSC; primary hepatic stellate cells.

^#^Calnexin is not commonly used as EV markers despite being found in EVs (Théry et al., 2018).

Immune modulation in the form of evasion and suppression represent a fundamental process whereby viruses establish persistent infection (**Figure 2**). Indeed, viruses have been able to co-evolve with their hosts successfully for centuries as obligate intracellular parasites using various immune evasion and suppression strategies. However, utilization of EVs as a mechanism for immune modulation by viruses is a relatively new concept put forward by various groups. It has been shown that EVs isolated using immunomagnetic separation from patients with chronic HBV infection were able to inhibit NK cell functions (**Table 2**) (Yang et al., 2017). Interestingly, these EVs were also able to transfer HBV rcDNA and HBV RNA (HBx and HBs/p) into NK cells from healthy donors when co-cultured (Yang et al., 2017). A similar finding was reported with HCV, where HCV-containing EVs from Huh-7.5.1 hepatocytes infected with HCV were able to induce expression of galectin-9 on monocytes (**Table 2**; **Figure 2**) (Harwood et al., 2016). Galectin-9 was previously found to be upregulated on Kupffer cells in livers of HCV-infected patients, which coincides with an elevated level of galectin-9 in chronic HCV patients. It was reported that galectin-9 promotes the expansion of Foxp3^+^ regulatory T cells (Tregs) and apoptosis of HCV-specific T cells (Mengshol et al., 2010), resulting in immunosuppression.

EVs harvested from HepAD38 expressing HBV pgRNA (using density gradient centrifugation) were able to immunosuppress monocytes by upregulating expression of programmed death-ligand-1 (PD-L1) (Kakizaki et al., 2018). PD-L1 is known to bind to PD-1 expressed on T cells and inhibits T cell activation and proliferation, which was evidenced by a decrease in expression of an early marker of lymphocyte activation, CD69 in that study (Kakizaki et al., 2018). Furthermore, microRNAs (miRNAs) associated with EVs have been implicated in the modulation of the host immune response by HBV. In particular, EV-associated miR-21 and miR-29a, which were isolated from pHBV-transfected HepG2 cells, downregulate IL-12 production and depress the immune response to HBV (**Table 2**) (Kouwaki et al., 2016). IL-12 is a known key regulator for Th1 differentiation and NK cell activation (Scharton-Kersten et al., 1995). Importantly, IL-12 also stimulates IFN-γ secretion from activated T and NK cells (Gately et al., 1998). The expression of miR-21 in the context of HCC development has also been shown to positively correlate with HCC progression (**Table 2**). Cao et al. found that EV- associated miR-21 positively regulates proliferation and migration of HCC cells, resulting in tumor growth and metastasis (Cao et al., 2019). In another study, Liu et al**. has shown that EV-associated miR-92a-2-5p isolated from THP-1 macrophages were able to increase liver cancer cell invasion by decreasing the expression of androgen receptors on cancer cells (Liu et al., 2020). It is noteworthy that the role of EV-associated miRNA in disease progression is, however, not restricted to immune cells in CLD. EVs derived from HCV-infected hepatocytes were reported to contain miR-19a that activated HSCs for differentiation into myofibroblasts (Devhare et al., 2017). It was found that miR-19a was highly upregulated in the sera of chronic HCV patients (Devhare et al., 2017).

Taken together, we propose that viruses move from the acute phase of infection to the chronic phase of infection by virtue of their biology, immune evasion mechanisms, and modulation of the host immune response. While the initial innate immune response provides the first line of defense against invading viruses such as HBV and HCV, its ability for viral clearance is limited (Szabo and Dolganiuc, 2008; Tang et al., 2018). As such, it is possible that EVs act as a sanctuary for viral components of HBV and HCV during the acute phase of infection where it would be difficult for virus-specific antibodies or immune cells to have access to them (Sanada et al., 2016). This provides the viruses with an excellent mode of transmission to uninfected cells, allowing viral persistence to be established (Yang et al., 2017), while the immune system continuously attempts to eliminate the viruses without any success. Persistent unproductive immune response results in an inflammatory microenvironment within the liver that is favorable for the development of liver fibrosis, cirrhosis, and HCC transformation (Ringelhan et al., 2017; Chen and Tian, 2019). Indeed, it would be interesting to test whether blocking the EV biogenesis pathway would decrease the risk for the development of liver cirrhosis and HCC since studies in cell culture systems have already shown that HCV titer is lowered when the release of EVs is blocked (Ramakrishnaiah et al., 2013; Shrivastava et al., 2013). To this end, it is important to emphasize that the current understanding of the role of EVs in viral-associated liver disease is incomplete and more research is required to bridge knowledge gaps.

#### The Role of Extracellular Vesicles as a Biomarker for Virus-Associated Chronic Liver Disease

EVs from blood and urine may be used as a potential non-invasive diagnostic tool for early detection of viral-associated CLD and HCC, which remain challenging to diagnose in part because most patients remain asymptomatic in early stages of disease pathogenesis. Of note, a large proportion (55-85%) of HCV patients have been reported to develop chronic hepatitis from the initial acute phase of the infection (Ringelhan et al., 2017). It is estimated that 20–30% of chronic HCV patients develop liver cirrhosis 15–25 years after infection (Lingala and Ghany, 2015) and 67–91% of these patients die from liver-associated causes such as HCC and liver failure if they do not receive timely antiviral therapy (Fattovich et al., 2002; Kobayashi et al., 2006; Toshikuni et al., 2009). Furthermore, poor prognosis of HCC is often associated with a late diagnosis of the disease. The 5-year survival rate for HCC is 12%, with a median survival of an estimated 6 to 20 months following diagnosis (McGlynn and London, 2011; McGlynn et al., 2015; Golabi et al., 2017). The 5-year survival rate increases to > 70% for patients if the diagnosis is made at an early stage (Tsuchiya et al., 2015).

HBV infection is currently detected *via* a serological assay for viral antigen, followed by a confirmatory real-time PCR test for viral DNA (Krajden et al., 2005; WHO, 2017). HCV is detected *via* a serological assay for viral antibody followed by confirmatory real-time PCR for RNA (Krajden et al., 2005; WHO, 2017). Subsequent staging of liver disease upon confirmation of viral infection involves assessment for clinical features of advanced liver disease/cirrhosis and non-invasive tests such as aspartate aminotransferase (AST)-to-platelet ratio index (APRI) and transient elastography (FibroScan) (WHO, 2017). However, clinical assessment, APRI and transient elastography have inherent limitations and failure rates; for instance, APRI is not liver-specific and does not discriminate intermediate fibrosis stages well, while transient elastography may have high failure rates and false positive results due to obesity, non-fasting state, acute hepatitis and inflammation, and inexperienced operators (Patel and Sebastiani, 2020).

In terms of diagnosis of HCC, it is unique cancer in that the diagnosis can be made solely on the basis of specific imaging characteristics of liver lesions using computer tomography imaging or magnetic resonance imaging without the need for liver biopsy (Cartier and Aubé, 2014). However, a definitive diagnosis of HCC in a non-cirrhosis disease background may still require an invasive liver biopsy. While liver biopsy remains the reference standard for the diagnosis of HCC, it is a costly procedure that only allows a small part of the liver to be examined and interpreted (Sumida et al., 2014; Sung et al., 2018). This increases the chance of sampling errors in heterogeneous solid liver tumor (Sung et al., 2018). Moreover, this procedure is associated with known morbidities, such as pain and complications. Therefore, it has been reserved for more complicated cases of HCC which cannot be definitively diagnosed using non-invasive imaging methods (Cartier and Aubé, 2014).

To circumvent the limitations associated with the current diagnostic regime for early detection of viral-associated CLD and HCC, various different types of “liquid biopsy” have been proposed as a potential diagnostic tool by several groups (Weis et al., 2019; Jeffrey et al., 2020). Liquid biopsies may contain various different combinations of analytes, circulating tumor cells, cell-free tumor DNA, mRNA, miRNAs, proteins, or metabolites (Mattox et al., 2019), as demonstrated for the diagnosis of HBV-derived HCC by Qu et al. (2019; Jeffrey et al., 2020). Weis and colleagues recently proposed in a pilot study the use of a serum miRNA panel comprising miR-122-5p, miR-486-5p, and miR-142-3p to discriminate HCV-derived HCC from mild disease and cirrhosis (sensitivity of 80%, a specificity of 95%, the negative predictive value of 82%, the positive predictive value of 74%, and overall accuracy of 78%) (Weis et al., 2019), although these miRNAs were not identified as EV-associated. More recently, there has been a growing interest in EVs as biomarkers for viral-associated CLD and HCC. An EV-based liquid biopsy is useful because EVs from diseased individuals often carry specific proteins, nucleic acids, and metabolites that can reveal the status of disease (Whiteside, 2017; Hoshino et al., 2020). Unlike standard liver biopsy, liquid biopsy is non-invasive and less costly to perform. Furthermore, as EVs contain molecules that are specific to a particular organ or disease, EV-based liquid biopsy can be optimized to be highly disease-specific. Such non-invasive tests also allow for a complementary or standalone test, providing rapid diagnosis or prognosis of diseases, such as the FDA approved EV-based biomarker for prostate cancer ExoDX (Tutrone et al., 2020).

Several EV-associated miRNAs such as miR-21, miR-221, and miR-222 have been linked to HCC (**Table 2**; **Figure 2**) (Meng et al., 2007; Varnholt et al., 2008; Yang et al., 2014; Sohn et al., 2015). Of interest, Tanaka et al**. reported a higher level of EV-associated miR-21 in the sera of cancer patients compared with healthy individuals (Tanaka et al., 2013). Furthermore, the expression of miR-21 was found to be significantly higher in the EVs than that in the EV-depleted sera of patients with HCC, which further suggests that the highly enriched EV-associated miR-21 could be a more reliable diagnostic parameter than free circulating miR-21 in these patients (**Table 2**) (Wang et al., 2014).

Given that viral-induced liver fibrosis in HCV-infected patients is the primary cause of viral-associated HCC, early detection of liver fibrosis could prove beneficial for the long-term surveillance of HCC. Specifically, EV-associated miR-214 and miR-199a-5p have been reported as potential fibrosis-related EVs in the liver (**Table 2**; **Figure 2**). In one study, downregulation of miR-214 was shown to increase connective tissue growth factor (CCN2), which drives fibrogenesis in activated primary murine HSCs and human HSCs, LX-2 (Chen et al., 2014). Similarly, it was found that miR-199a-5p also works in a similar fashion as miR-214, resulting in reduced levels of EV-associated miR-199a-5p in fibrotic mouse livers and activated primary murine HSCs (Chen et al., 2016). Furthermore, Lambrecht et al**. investigated the use of EV-associated miR-122, miR-192, and miR-200b for the early staging of hepatic fibrosis in chronic HBV and HCV patients and reported increased levels of all three miRNAs in total plasma of early-stage chronic patients (Lambrecht et al., 2017).

#### Extracellular Vesicles as Therapeutics for Chronic Liver Disease

In recent years, EVs have attracted enormous attention as a delivery system for therapeutic molecules. Unlike liposomes and nanoparticles, the current preferred carrier choices for drug delivery, EVs are naturally occurring nano-sized biological carriers in a eukaryotic system (Akuma et al., 2019). Due to their cellular origin and small size, EVs are biocompatible, non-toxic, and less immunogenic than liposome-based drug delivery, which make them better candidates than the artificially made liposomes and nanoparticles as drug delivery agents (Wu et al., 2019). Furthermore, they are able to carry a wide range of biological molecules across biological barriers such as the blood-brain barrier, increasing the bioavailability of the molecules in the biological system and reducing the dosage required for therapeutic benefit (Akuma et al., 2019). The structural composition of EVs also provides a protected enclosed space, making it an ideal delivery carrier for gene therapy, anti-fibrotic and cancer treatments (Wang et al., 2019). However, it is worth mentioning that the use of EVs as a drug delivery system is not without limitations. The feasibility to scale-up primary cell culture to obtain sufficient EVs for clinical use is an important consideration and also a significant challenge (Raimondo et al., 2019). While tumorigenic or immortalized cell lines offer an easier alternative to primary cells for large scale production of EVs, EV-derived from cancer cell lines may inherently pose a risk of undesirable horizontal gene transfer which may have dire consequences in the event that oncogenes are transferred into EVs (Raimondo et al., 2019). Isolating and purifying EVs can also be extremely costly, labor-intensive and time-consuming, although the technologies have been continuously improved (Soekmadji et al., 2020).

At present, one of the challenges with the treatment of HCC is the lack of effective therapeutic agents which can successfully improve the overall survival of HCC patients. There are currently six approved drugs available for treatment of HCC with only moderate success (Jindal et al., 2019). Due to the heterogeneity of the patient population and increasing number of advanced HCC patients showing drug resistance (Niu et al., 2017; Namee and O’Driscoll, 2018; Wang et al., 2019), there is an urgent need to develop innovative therapy for HCC (Jindal et al., 2019). Takahashi *et al*. found a role of EV-associated long non-coding RNAs, linc-ROR, in HCC cells which mediate chemoresistance through attenuation of drug-induced apoptosis and inhibition of p53 expression (Takahashi et al., 2014). In light of the need for new and effective cancer therapy, EVs have shown great promise in delivering a common chemotherapeutic agent, paclitaxel to treat multiple drug resistance cells (Kim et al., 2016). Kim et al. demonstrated that there was at least a 50-fold increase in cytotoxicity in drug-resistant cells using the EV-based treatment (Kim et al., 2016). Several studies have also utilized EVs to deliver anti-tumor therapeutics such as methotrexate and doxorubicin to destroy HCC cells (Tang et al., 2012; Tian et al., 2014). Immunotherapy involving EVs as carriers is a promising alternative treatment for HCC. Rao et al**. demonstrated a significant reduction in tumor growth in HCC mice treated with HCC-derived EVs which were able to elicit a strong dendritic cell-mediated anti-tumor immune response (**Figure 2**) (Rao et al., 2016). In addition, the tumor microenvironment was also found to have substantially higher levels of infiltrating CD8^+^ T cells and inflammatory cytokines (Rao et al., 2016).

Viral-induced liver fibrosis and cirrhosis is one of the major risk factors for the development of HCC. Therefore, slowing or halting the progression of hepatic fibrosis will likely be beneficial in reducing the risks for malignancy transformation. Chen *et al*. found that EV-associated miR-199a-5p derived from quiescent HSCs was able to reduce the expression of fibrogenic genes and proteins in activated HSCs (**Figure 2**) (Chen et al., 2016). Furthermore, EVs released from adipose-derived mesenchymal stem cells (MSC) transfected with miR-122 were able to inhibit activation and proliferation of HSCs. Interestingly, these MSC-derived EVs were also able to inhibit fibrosis in the livers of mice exposed to carbon tetrachloride (Lou et al., 2017). Thus, EVs may have the potential to be used as therapeutics to treat CLD, with further research in this area clearly warranted.

## Conclusions and Future Directions

EVs represent a novel class of nanoparticles, which could be involved in the pathogenesis and progression of CLD. Indeed, evidence gathered from recent studies has shed light on the role of EVs in viral-associated CLD and HCC. EVs have emerged as an important, yet poorly understood mechanism utilized by viruses and the host immune system for disease pathogenesis of viral-associated CLD and HCC. While there is evidence that EVs have the potential to play a role as non-invasive diagnostic tools for early detection of disease and as carriers for therapeutics, a more thorough understanding of EV biogenesis and disease pathogenesis and better, internationally standardized technologies for the isolation and enrichment of EVs are warranted before they can be widely used in the clinic for the treatment of CLD.

## Author Contributions

All authors contributed to the article and approved the submitted version.

## Conflict of Interest

The authors declare that the research was conducted in the absence of any commercial or financial relationships that could be construed as a potential conflict of interest.
